# Solid-state stability study of meropenem – solutions based on spectrophotometric analysis

**DOI:** 10.1186/1752-153X-7-98

**Published:** 2013-06-08

**Authors:** Judyta Cielecka-Piontek, Magdalena Paczkowska, Kornelia Lewandowska, Boleslaw Barszcz, Przemyslaw Zalewski, Piotr Garbacki

**Affiliations:** 1 Department of Pharmaceutical Chemistry, Faculty of Pharmacy, Poznan University of Medical Sciences, Grunwaldzka 6, Poznań, 60-780, Poland; 2 Department of Molecular Crystals, Institute of Molecular Physics Polish Academy of Sciences, Smoluchowskiego 17, Poznań, 60-179, Poland

**Keywords:** Meropenem, Derivative spectroscopy, FT-IR spectroscopy, Raman-spectroscopy, Degradation, Solid state

## Abstract

**Background:**

B-Lactam antibiotics are still the most common group of chemotherapeutic drugs that are used in the treatment of bacterial infections. However, due to their chemical instability the potential to apply them as oral pharmacotherapeutics is often limited and so it is vital to employ suitable non-destructive analytical methods. Hence, in order to analyze such labile drugs as β-lactam analogs, the application of rapid and reliable analytical techniques which do not require transferring to solutions or using organic solvents, following the current *green* approach to pharmaceutical analysis, is necessary. The main objective of the present research was to develop analytical methods for the evaluation of changes in meropenem in the solid state during a stability study.

**Results:**

The UV, FT-IR and Raman spectra of meropenem were recorded during a solid-state stability study. The optimum molecular geometry, harmonic vibrational frequencies, infrared intensities and Raman scattering activities were calculated according to the density-functional theory (DFT/B3LYP method) with a 6-31G(d,p) basis set. As the differences between the observed and scaled wavenumber values were small, a detailed interpretation of the FT-IR and Raman spectra was possible for non-degraded and degraded samples of meropenem. The problem of the overlapping spectra of meropenem and ring-containing degradation products was solved by measuring changes in the values of the first-derivative amplitudes of the zero-order spectra of aqueous solutions of meropenem. Also, molecular electrostatic potential (MEP), front molecular orbitals (FMOs) and the gap potential between highest occupied molecular orbital (HOMO) and lowest unoccupied molecular orbital (LUMO) were determined.

**Conclusions:**

Based on the findings of this work, it appears possible to use time-saving and reliable spectrophotometric analytical methods, supported by quantum-chemical calculations, for solid-state stability investigations of meropenem. The methods developed for this study may be considered a novel, *green* solution to pharmaceutical analysis of labile drugs – an alternative for the recommended chromatographic procedures.

## Background

The chemical instability of drugs may reduce pharmacotherapeutic possibilities and affect the selection criteria when deciding on analytical methods for the quality control of labile medicines. In that group of drugs, the problem of the susceptibility of β-lactam antibiotics to degradation in solutions and in the solid state has often been reported [1-3]. Since oral administration of antibiotics is not possible in the case of some β-lactam analogs, the remaining solution is the parenteral route, which involves the risk of unwanted side effects [4,5]. In view of the significant lability of β-lactam analogs, the application of suitable analytical methods is of utmost importance. The quality control of drugs in the solid state is expected to rely on methods ensuring minimization of sample transformation, for example elimination of sample derivatization or limitation of reactions with chemical reagents. As it has been established, organic solvents affect the rate of degradation of β-lactam antibiotics as well as the formation of degradation products [6-8]. Therefore, the methodology of studying β-lactam antibiotics in solutions is required to eliminate such reagents that provide inconsistent environments.

**Figure 1:**
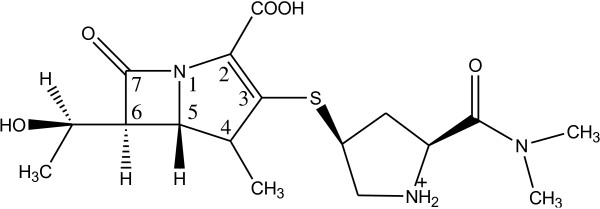
Chemical strucutre of meropenem.

Among the β-lactam antibiotics, carbapenems are analogs that are especially susceptible to enzymatic and chemical degradation [9]. Meropenem is a carbapenem analog in which the introduction of a methyl group at C4 solved the problem of enzymatic instability (Figure 1). What still needs a solution is the lack of chemical stability of meropenem, a problem which may be addressed by finding control methods ensuring therapeutic safety and effectiveness [10,11]. Meropenem is the first methylcarbapenem analog introduced into therapeutic use [12,13] characterized by activity against a wide range of Gram-negative and Gram-positive aerobic and anaerobic bacteria [14,15]. Recent studies proved meropenem to be especially efficient in the treatment of tuberculosis, caused by *Mycobacterium tuberculosis,* after connection with clavulanic acid, an inhibitor of β-lactamases [16,17].

Therefore, in light of the therapeutic potential of meropenem, including its bactericidal activity against resistant strains, it is vital to develop methods of quality control able to take into account its significant susceptibility to degradation. Meropenem is known to be very unstable in aqueous solutions and in the solid state [18]. Depending on affecting factors and the form of meropenem, different degradants are formed. For example, during the degradation of meropenem in acidic solutions degradation products with an open β-lactam ring are formed whereas thermal degradation of meropenem leads to the formation of 4-methyl-3-(1H-pyrrol-3-ylsulfanyl)-5H-pyrrole-2-carboxylic acid. Mendez *et al.* suggested that the modification of the side chain of meropenem in the direction of a pyrrolic-ring degradation product is a result of decarboxylation and aromatization of the pyrrolidine ring and hydrolysis of the β-lactam ring, considered an intermediate stage of degradation [19,20]. Also, it was observed that during solid-state stability studies of carbapenem analogs different degradants were formed during thermolysis as a consequence of the highly strained fused ring system [21,22].

The most common techniques for the determination of meropenem during stability studies are based on chromatography. Due to the insolubility of carbapenems in organic solvents, only reversed-phase (RP) liquid chromatography is used in the analysis of meropenem [23]. Since the degradation of β-lactam analogs is known to be additionally affected by solvolysis, it appears justified to eliminate the use of organic solvents [24]. Therefore, in order to ensure the reliability of determination, it is vital to use such analytical methods that do not require the application of organic solvents or the placement of samples in media other than target intravenous solutions during studies of meropenem in the solid state. The analytical procedures that overcome those problems are spectral methods. They allow a rapid analysis of meropenem in the solid state (FT-IR, Raman spectroscopy) and in aqueous solutions, relying exclusively on dissolution in water samples (UV). For the determination of carbapenems, including meropenem, only first-derivative, first-derivative spectrum ratio and third-derivative UV spectroscopies were used, which permitted eliminating interferences originating from degradation products with an open β-lactam ring and dimers [20,25,26] formed in aqueous solutions. A literature review has not demonstrated the use of spectroscopic methods as an alternative mode of quality control of meropenem in the solid state.

The main objective of the present study was to develop and apply spectrophotometric methods for the quality control of meropenem in the solid state, which involved investigating vibrations within the bonds in a meropenem molecule and their changes during degradation by using spectral methods (FT-IR, Raman spectroscopy) on the basis of chemical-quantum calculations and developing a selective analytical method for the determination of changes in meropenem concentrations in the solid state with the use of water instead of organic solvents.

## Experimental

### Substances and spectroscopic measurements

The meropenem reference standards (purity > 98%) were supplied by Pharmachem International Co., (China). The commercial preparation of meropenem, Meronem®, contained meropenem 500 mg and sodium carbonate 140 mg.

The first derivatives of ultraviolet spectra of meropenem were recorded by using a UV–VIS Lambda 20 (PerkinElmer) spectrophotometer equipped with 1.0 cm-in-width quartz cells and controlled via the UV WinLab software. Water was used as solvent. The vibrational infrared spectra of meropenem were recorded between 400 and 7000 cm^-1^ in powder, at room temperature, with an FT-IR Bruker Equinox 55 spectrometer equipped with a Bruker Hyperion 1000 microscope. The Raman scattering spectra were obtained with a LabRAM HR800 spectrometer (Horiba Jobin Yvon) with laser excitation λ_exc_ = 633 nm (He-Ne laser). In each case the power of the laser beam at the sample was less than 1mW to avoid damaging the sample. High quality pure water was prepared using an Exil SA 67120 purification system (Millipore).

### Stability studies

For stability studies, 5 mg samples of meropenem were weighed into 5 ml vials. To evaluate their stability at increased air humidity, they were placed in heat chambers at 313 K in desiccators containing saturated solutions of sodium chloride (relative humidity (RH) ≈ 76.4%). The changes of meropenem concentration were studied after 15; 30; 45; 60; 75; 90; 105 and 120 minutes of degradation. To evaluate the stability of meropenem in dry air, the vials were immersed in a sand bath placed in heat chambers at 323 K. The sand was first dried, and then kept at 323 K with preventing the absorption of water vapor from the environment. The changes of meropenem concentration were studied after 2.5; 5; 7.5; 10; 12.5; 15; 17.5; and 20 hours of degradation.

The UV method was validated according to the International Conference on Harmonization Guidelines [27] in terms of linearity, precision, accuracy, and limits of detection and quantitation for meropenem were established.

### Theoretical calculations

The derivative spectrophotometric method was based on the transformation of the zero-order spectrum of meropenem into its first derivative (∆A/∆λ) by using the UV WinLab software. In order to interpret the experimental results of IR absorption and Raman scattering, quantum-chemical calculations were performed by using the Gaussian 03 package [28]. The GaussView software was utilized to propose the initial geometry of the investigated molecules and to visually inspect the normal modes. The molecular geometries were optimized by means of a density functional theory (DFT) method with the B3LYP hybrid functional and a 6-31G(d,p) basis set.

## Results and discussion

The FT-IR and Raman spectra of non-degraded and degraded samples of meropenem obtained in this study allowed characterization of molecular vibrations as well as changes in its structure resulting from degradation during storage in the solid state. The calculated IR and Raman scattering spectra, obtained by means of the density functional theory, were used as reference. The application of the first derivative of the zero-order UV spectra permitted determination of meropenem in the presence of 4-methyl-3-(1H-pyrrol-3-ylsulfanyl)-5H-pyrrole-2-carboxylic acid, which is a degradation product that can form under the recommended storage conditions for meropenem preparations [20]. Additionally, the FMOs and the MEP of meropenem were determined based on quantum-chemical calculations. The following sections discuss spectral analysis and the findings of theoretical calculations.

### Vibrational assignments

**Figure 2:**
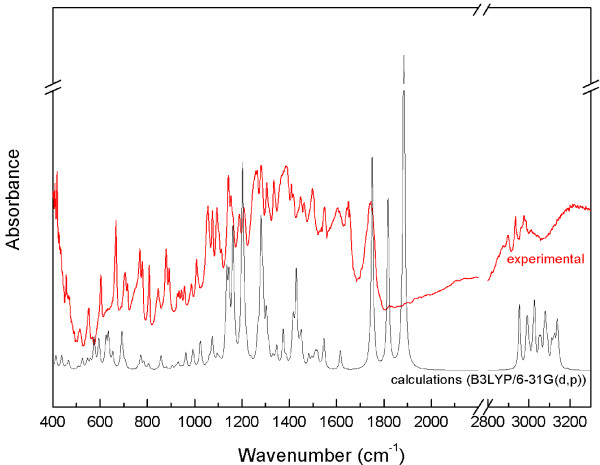
Experimental and calculated (B3LYP/6-31G (d,p)) FT-IR spectra for meropenem.

**Figure 3:**
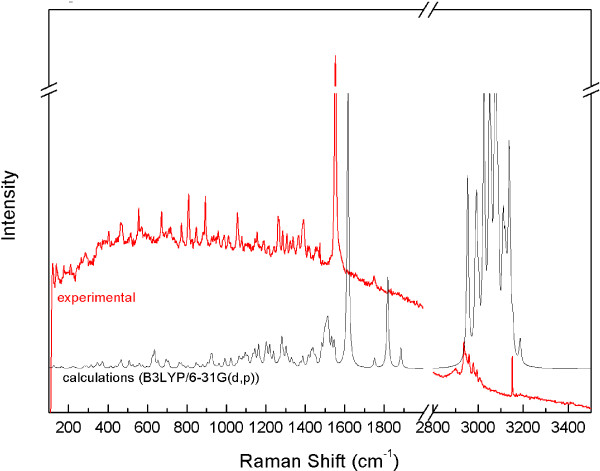
Experimental and calculated (B3LYP/6-31G (d,p)) Raman spectra for meropenem.

A number of bands corresponding to the vibrations of the β-lactam and pyrrolidine 4:5 bicyclic fused rings and of certain substituents were identified in the IR absorption and Raman scattering spectra of meropenem. Let us start with the bands located at 653 and 690 cm^-1^. These modes also contained components corresponding to the bending vibrations of the C-O-H bond in a carboxyl group. In the experimental IR spectra they were located at 668 and 705 cm^-1^. The bands corresponding to the bending vibrations of the carboxyl group C-O-H bond were also observed at 771 and 768 cm^-1^ in the calculated and experimental IR spectra, respectively. The bending vibrations of the C-O-H bond were observed at 1202 and 1188 cm^-1^ (calculated and experimental IR and Raman spectra, respectively), too. This mode also showed components corresponding to the stretching vibration of the C-N bond in the β-lactam ring. The bands related to the stretching vibrations of the C-N bond in the β-lactam ring were also observed in the calculated spectra at 1135 and 1417 cm^-1^. In the experimental spectra, they were located at 1143 and 1388/1391 cm^-1^ (IR/Raman, respectively). The band at 1135 cm^-1^ was corresponding also to the wagging and twisting vibrations of the C-H bonds present in the entire structure. The band at 1417 cm^-1^ was complex and composed of many normal modes of vibrations. This mode had additional components corresponding to the stretching vibrations of the C-C bond between the pyrrolidine ring and the carboxyl group and to the scissoring vibrations of the C-H bond in the methyl group in the *trans*-hydroxyethyl group at C2. The band related to the stretching vibrations of the C-C bond in the β-lactam ring was also observed at 994/989 cm^-1^ in the calculated/experimental Raman scattering spectra. The range 1600–1900 cm^-1^ in the calculated spectra exhibited distinct bands related to the stretching vibrations of the C=C and C=O bonds. The band associated with the stretching of the C=C bond in the β-lactam and pyrrolidine 4:5 bicyclic fused rings was located in the calculated spectra at 1616 cm^-1^. In the experimental spectra, they were observed at 1549 and 1553 cm^-1^ in the IR and Raman spectra, respectively. The stretching vibrations of the C=O bond in the β-lactam ring were located at 1887/1749 cm^-1^ (calculated/experimental IR spectra). Lower wavenumbers in the calculated spectra (1751 and 1818 cm^-1^) revealed bands corresponding also to the stretching vibrations of the C=O bonds but in the *trans*-hydroxyethyl and carboxyl substituents. In the experimental IR spectra a split was observed into those bands and bands related to the vibrations at 1749, 1604 and 1651 cm^-1^, which resulted from the fact that the bonds located in the external part of meropenem molecules were likely to be affected by intermolecular interaction. Fairly distinct bands related to the breathing of the pyrrolidine ring at 695/715 cm^-1^ in the calculated/experimental IR spectra were located. That mode also contained components related to the bending vibrations of the C=O bond in the dimethylcarbamoyl group situated closest to the pyrrolidine ring. A band corresponding to the breathing of the pyrrolidine ring was identified in the theoretic Raman spectrum at 784 cm^-1^. In contrast, the stretching vibrations of the C-N bond between the nitrogen atom and the methyl groups in the dimethylcarbamoyl substituents in the experimental spectra were shifted to 810 cm^-1^. A greater band shift was observed for the bands located in the calculated Raman spectra at 991 cm^-1^, which was related to the stretching vibrations of the C-C bond of the β-lactam ring, especially between the carbonyl group and the C-N bond in that ring whereas between the nitrogen atom and the methyl group in the dimethylcarbamoyl substituents the stretching vibrations of the C-N bond were identified at 1289 cm^-1^ in the calculated spectra. In the experimental IR/Raman those bands were located at 1265/1263 cm^-1^. The stretching vibrations of the C-N bond in the dimethylcarbamoyl substituents were also observed at 1430/1388 cm^-1^ in the calculated/experimental IR spectra. Fairly distinct bands associated with the stretching vibrations of the C-C bond in the pyrrolidine ring as well as the wagging and twisting vibrations of the C-H bonds at the β-lactam ring were located at 1049 cm^-1^ in the calculated and at 1056 cm^-1^ in the experimental spectrum. The band corresponding to the stretching of the C-C bond present in the β-lactam and pyrrolidine 4:5 bicyclic fused rings and between the carbon atom in the carboxyl group was observed at 1330/1307 cm^-1^ in the calculated/experimental Raman spectra. Clearly visible bending vibrations of the O-H bonds in the carboxyl group were observed at 575/603 and 635/668 cm^-1^ in the calculated/experimental spectra, respectively. Many bands corresponding to the vibrations of the C-H bonds in the alkali substituents were found in the Raman and IR spectra. The bands related to the wagging and twisting vibrations of the C-H bonds in the *trans*-hydroxyethyl group and in the β-lactam and pyrrolidine 4:5 bicyclic fused rings were located in the experimental IR spectrum at 1075 and 1335 cm^-1^. The stretching vibration of the C-H bonds in that group occurred at 2961 and 2997 cm^-1^ in the experimental Raman scattering spectrum. A band associated with the stretching vibrations of the C-H bonds in the dimethylcarbamoyl group was identified at 2900 cm^-1^. In the IR and Raman scattering spectra, bands related to the scissoring and wagging vibrations of the C-H bonds in the dimethylcarbamoyl group were located between 1440–1500 cm^-1^. Bands corresponding to the twisting vibrations of the C-H bonds in the same part of the molecule were observed at 1143/1147 cm^-1^ in the calculated/experimental Raman spectra and at 1160/1153 cm^-1^ in the calculated/experimental IR spectra. Figures 2 and 3 present the experimental and calculated infrared and Raman scattering spectra of meropenem, respectively - the most important of which are listed in Table 1.

**Table 1:** Comparison of observed and calculated vibrational modes of meropenem before degradation

**IR calc/exp**	**Raman calc/exp**	**Band assignment**
**Wavenumber (cm**^**-1**^**)**		
**575/603**		O-H *b* in carboxylic group
**635/668**		O-H *b* in carboxylic group
**653/668**		def. pyrrolidine ring + C-O-H *b* in carboxyl group
**690/705**		def. pyrrolidine ring + C-O-H *b* in carboxyl group
**695/715**		breathing β-lactam ring + C=O *b* in dimethylcarbomoyl group
**771/768**		carbonyl group *b* in carboxylic group
	784/810	breathing β-lactam ring + C-N *s* between N and methyl group in dimethylcarbomoyl group
	991/959	C-C between β-lactam and dimethylcarbomoyl group + C-N in β-lactam
	994/989	C-C *s* in basis ring + CH_3_ w at pyrrolidine ring
**1049/1056**	1049/1056	C-C in β-lactam ring + CH *t*/*w* in *trans*-hydroxyethyl group
**1074/1075**		CH *t/w* in *trans*-hydroxyethyl group
**1135/1143**		CH *t/w* + C-N *s* in pyrrolidine ring
	1143/1147	CH_2_*t* and methyl *t* in dimethylcarbomoyl group
**1160/1153**		CH *t* in CH_2_ and methyl group in dimethylcarbomoyl group
**1202/1188**	1202/1188	C-N *s* in pyrrolidine ring + C-O-H *b* in carboxyl group
	1221/1210	C-H *w* at β-lactam ring
**1289/1265**	1289/1263	C-N *s* at 4 CH_3_ + C-H *w*
	1330/1307	C-C *s* between pyrrolidine ring and C at COH group
**1375/1335**		C-H *w* in CH in *trans*-hydroxyethyl group + CH w in pyrrolidine ring
**1417/1388**	1417/1391	C-C *s* between carboxyl group and pyrrolidine ring + C-N *s* in basis ring + C-H *sc* in CH_3_ in *trans*-hydroxyethyl group and methyl group
**1430/1388**		C-N *s* between CO and CH_3_ in dimethylcarbomoyl group
	1438/1421	C-H *sc* in dimethylcarbomoyl group
	1482/1459	C-H *w* in dimethylcarbomoyl group
	1513/1466	C-H *s*
**1519/1448**		CH3 *w/sc* in dimethylcarbomoyl group
**1548/1500**		CH3 *w/sc* in dimethylcarbomoyl group
**1616/1549**	1616/1553	C=C *s* in pyrrolidine ring
**1751/1749, 1604**		C=O *s* in *trans*-hydroxyethyl group
**1818/1749, 1651**	1818/1749	C=O *s* in carboxyl group
**1887/1749**		C=O *s* in pyrrolidine ring
	3020/2939	C-H *s* in dimethylcarbomoyl group
	3027/2947	C-H *s* in dimethylcarbomoyl group
	3052/2961	C-H *s* in *trans*-hydroxyethyl group group
	3137/2997	C-H *s* in methyl group

Vibrational modes: *s* stretching, *b* bending, *w* wagging, *sc* scissoring, *r* rocking, *t* twisting.

**Figure 4:**
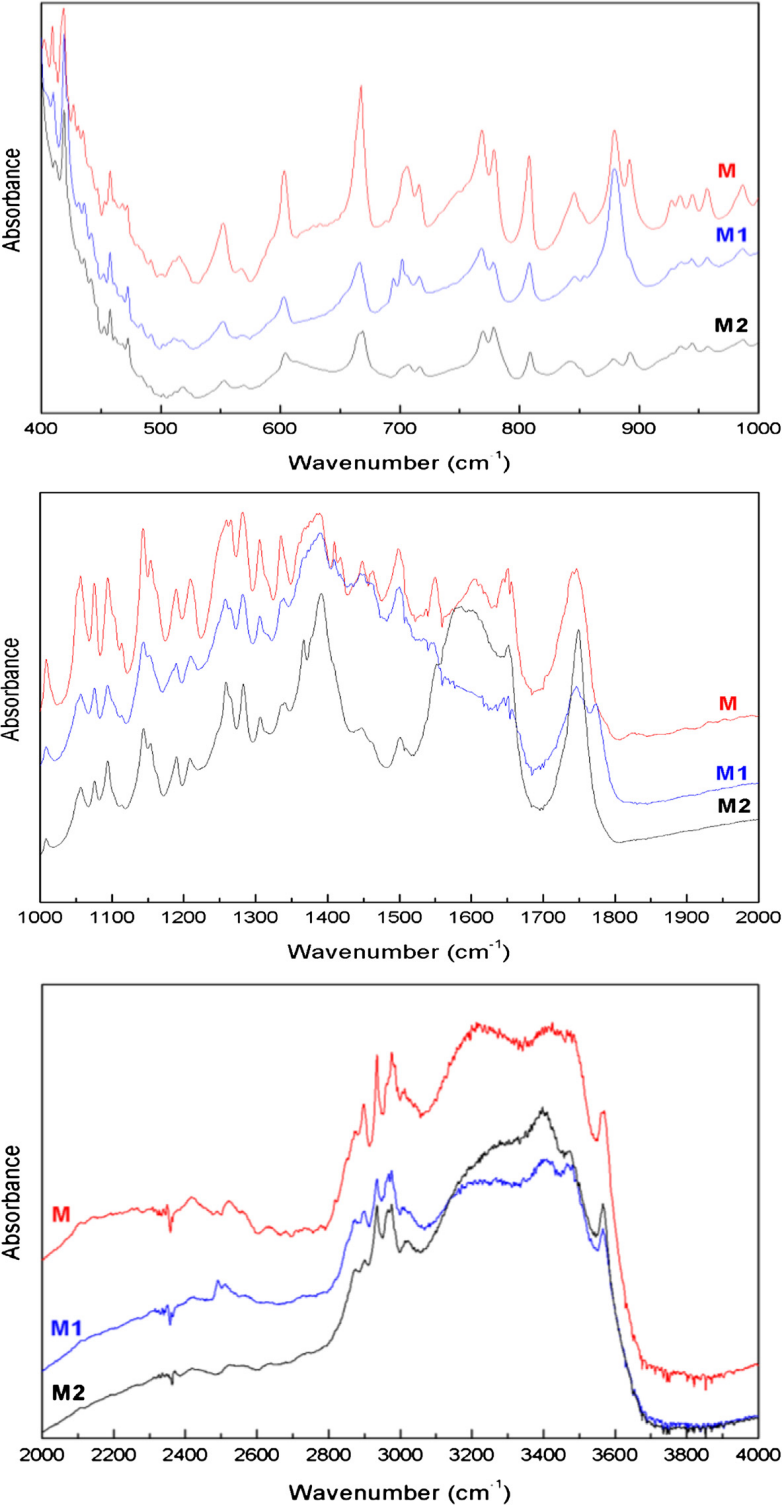
**Experimental FT-IR spectra for non-degraded and degraded meropenem (M - non-degraded sample; M**_**1 **_**- degraded samples at 50°C, RH = 0%, t = 5 h; M**_**2 **_**- degraded samples at 40°C, RH = 76.5%, t = 1 h).**

The next stage of this work was aimed at evaluating the suitability of FT-IR and Raman spectroscopies for the assessment of changes in the structure of meropenem as a consequence of exposure to temperature and humidity. The degradation of meropenem at increased relative air humidity (simulation of storage in a damaged container) and at increased temperature at RH = 0% (simulation of storage in an airtight container) was analyzed during a stability study (RH ≈ 76.4%, T= 313 K, t = 1.0 h and RH = 0%, T = 323 K, t = 5.0 h). It was found that by analyzing the FT-IR spectra of meropenem it was possible to assess changes in the structure of its samples at increased temperature, at RH = 0%. Based on a comparison of the spectra of non-degraded samples, using chemical-quantum calculations to identify vibrations, the most distinct alterations were observed in the ranges 660–700 cm^-1^, 850–900 cm^-1^, and 1350–1800 cm^-1^ (Figure 4). Regarding changes in the molecular structure of meropenem associated with the cleveage of bonds in the structure of the β-lactam and pyrrolidine 4:5 bicyclic fused rings, their manifestations included an increase in band intensity at 879 cm^-1^, changes in the shapes of the bands and in the ratios of the intensities between the bands in the range 1350–1800 cm^-1^, changes in the band at 1749 cm^-1^ corresponding to the stretching vibrations of the C=O bond in the β-lactam ring as well as the occurrence of a band at 695 cm^-1^ in samples degraded at T = 323 K, RH = 0%. The changes at 1500–1600 cm^-1^ could be explained by the formation of a new bond between the nitrogen and the hydrogen atoms in the pyrrolidine ring. Also, the intra- and inter-molecular impact of the open β-lactam ring might be responsible for the degradation process.

By analyzing changes in the FT-IR spectra of meropenem exposed to increased relative air humidity (RH = 76.4%, T = 40°C) it was possible to determine a cleavage of the β-lactam ring. The observed changes were not characteristic and were expressed by the appearance of bands at 1747 and 1748 cm^-1^ at increased temperature and RH = 0% and at increased relative air humidity, respectively.

The identification and evaluation of qualitative changes in meropenem that may occur during storage in commercial packaging based on analyzing the FT-IR spectra of that carbapenem analog may be considered a time-efficient, cost-effective and reliable analytical tool.

**Figure 5:**
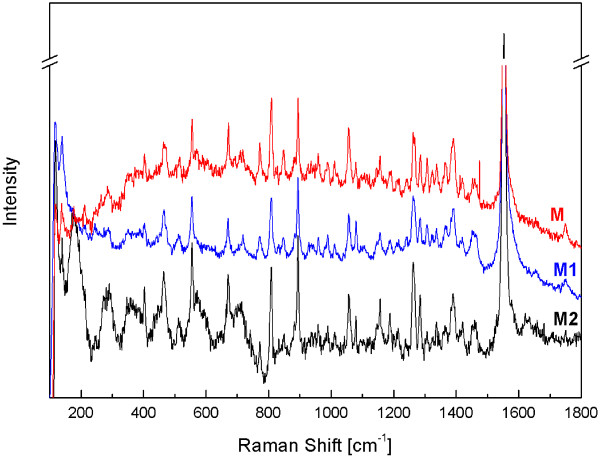
**Experimental Raman spectra for non-degraded and degraded meropenem (M - non-degraded sample; M**_**1 **_**- degraded samples at 50°C, RH = 0%, t = 5 h; M**_**2 **_**- degraded samples at 40°C, RH = 76.5%, t = 1 h).**

The identification of meropenem based on the analysis of Raman spectra may be recommended as an alternative method but only for non-degraded samples as the vibration bands of degradation products are not sufficiently distinct in Raman spectra, probably due to the fact meropenem contains covalently polarized bonds (Figure 5).

### UV spectral studies

**Figure 6:**
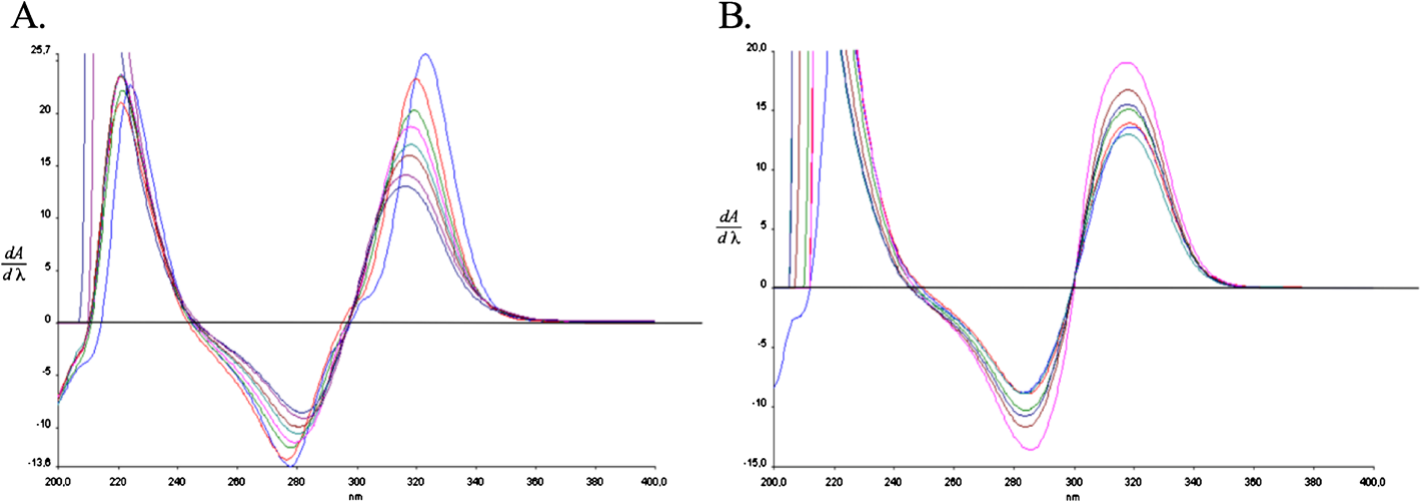
First-derivative spectra of meropenem during degradation in dry air at T=50°C, t=0; 2.5; 3.0; 7.5; 10.0; 12.5; 15.0; 17.5; 20 h) (A) and at increased relative humidity at RH=76.5% , T=40°C, t=0; 15; 30; 45; 60; 75; 90; 105; 120 min (B).

The zero-order absorption spectra of meropenem and of its solid-state degradation products overlapped whereas their first-derivative spectra permitted elimination of that problem. As a result of applying derivative spectrophotometry, the linear differences in the values of peak amplitudes of meropenem (at a zero-crossing wavelength of 307 nm) in response to changes in the concentration of meropenem were determined (Figure 6). In the first-derivative spectra of degraded samples of meropenem under the influence of increased temperature in dry air, a bathochromic effect was observed. A shift of bands in the UV spectrum related to the formation of degradants was also observed when evaluating changes in the FT-IR spectra of samples of degraded meropenem under the same conditions. Elragehy *et al.* reported application of UV spectroscopy for the determination of meropenem in the presence of degradation products formed in solutions. The problem of overlapping open-ring degradation products as the main degradants was solved by employing first-derivative, first-derivative spectrum ratio and bivariate analyses. The hypsochromic effect of zero-order spectra was reported [20].

By achieving the desired selectivity of determination of meropenem after applying first-derivative spectrophotometry, it was possible to analyze it quantitatively. The calibration curve was described by the equation y=(111,30 ±2,27) × (λ = 320 nm). The *b* values calculated from the equation y = *a*x + *b* were not significant. A good intra-day repeatability was determined (1.64%–2.29%) for the three levels (80%, 100% and 120%) of nominal concentration of meropenem in the linearity range 25–131 μg/mL. Inter-day repeatability also had acceptable values. The percentage recovery of meropenem ranged from 99.9% to 101.3% in a pharmaceutical dosage form. The limits of detection and quantitation, describing the smallest concentration of an analyte that can be reliably measured by an analytical procedure, were 3.58 μg/mL and 11.0 μg/mL, respectively.

The application of first-derivative spectrophotometry for the determination of meropenem may be a solution to the problem of overlapping bands. The achievement of required validation parameters and the advantages of spectrophotometric determination of meropenem make this method suitable for a routine analysis of that carbapenem analog, allowing its determination in the presence of solid-state degradation products.

### DFT studies

The analysis of the optimized geometry of meropenem indicated that the intraring stress is a result of the fusion of the heterocyclic moieties with the substituents, which can be spatial obstacles to each other. Such molecular configuration of meropenem ensures its binding with PBP (penicillin-binding protein) enzymes that is responsible for antibacterial activity. At the same time that configuration determines the significant susceptibility to degradation of meropenem under the conditions of acid–base hydrolysis and in the presence of oxidizing factors as well as during thermolysis.

**Figure 7:**
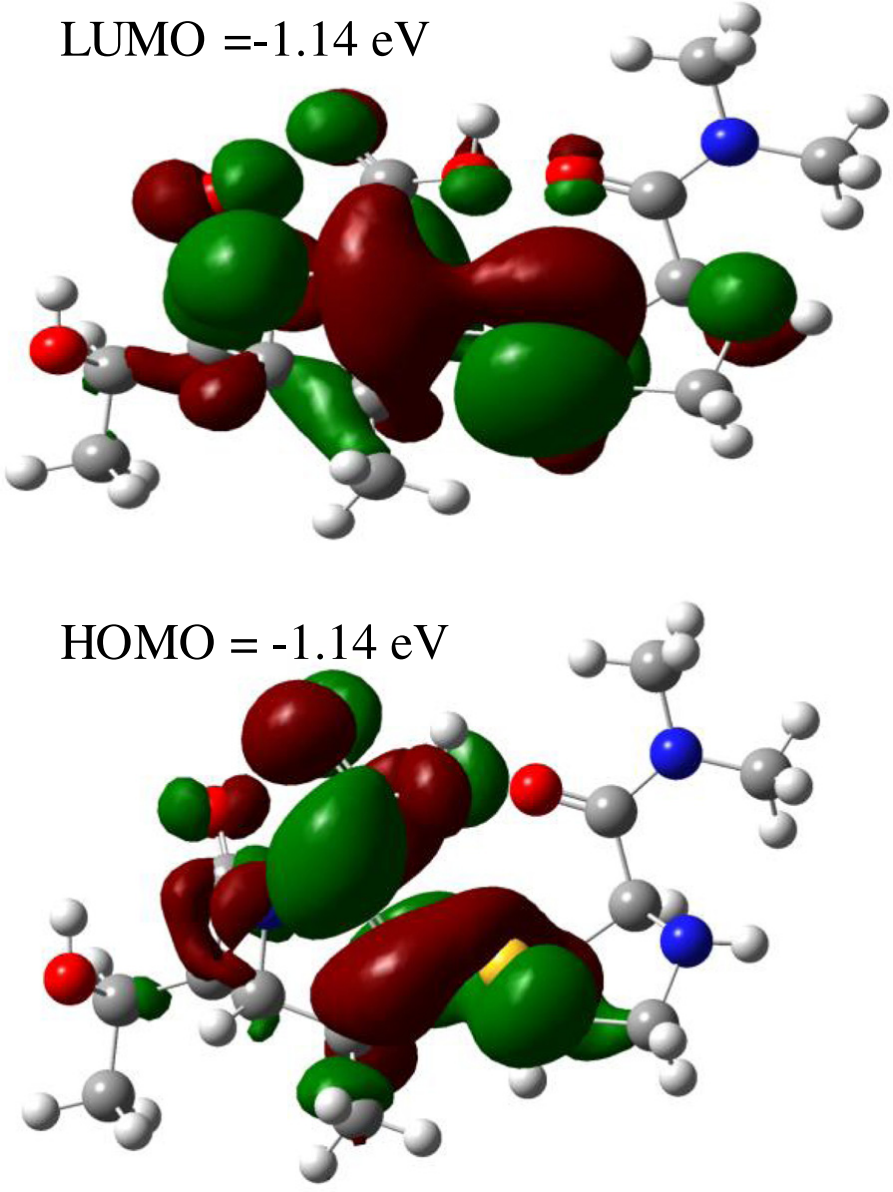
Atomic orbitals composition for the frontier molecular orbital for meropenem.

The localization of charge density on the frontier molecular orbitals, calculated with the B3LYP hybrid functional and a 6-31G(d,p) basis set, demonstrates a similar localization for the lowest unoccupied molecular orbital and the highest occupied molecular orbital. For both the HOMO and LUMO, the charge density was localized on the β-lactam and pyrrolidine 4:5 bicyclic fused rings and the carboxylic and carbonyl groups (Figure 7). Since the FMOs are the main orbitals involved in reactivity, the 4:5 bicyclic fused rings of meropenem may be proposed as the main areas where acceptor-donor electron reactions occur. The low value of HOMO-LUMO gap energy for meropenem (4.25 eV) confirms its significant susceptibility to degradation.

**Figure 8:**
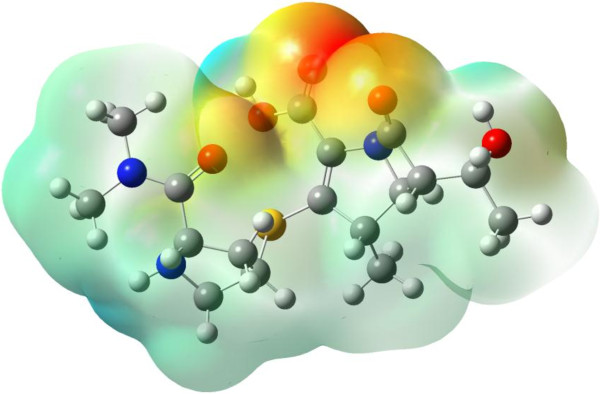
Molecular electrostatic potential map (MEP) for meropenem.

In order to predict reactive sites for electrophilic and nucleophilic attack in meropenem, the MEP was also established (Figure 8). The different values of electrostatic potential on the surface are represented by colors. The positive (blue) regions of MEP show electrophilic while the negative (red) areas nucleophilic reactivity. In meropenem, the most pronounced are the negative regions, localized on the carboxylic and carbonyl groups, that indicate possible sites for nucleophilic activity.

## Conclusions

The proposed methods (UV, FT-IR and Raman spectroscopy) for the quality assessment of meropenem and its determination in the presence of degradation products formed during solid-state storage may be considered superior to commonly applied chromatographic techniques. The spectral and theoretical studies conducted in this work permitted evaluation of molecular changes in meropenem and characterization of the inter- and intra-molecular interactions observed during its storage in the solid state. The analysis of the frontier molecular orbitals and the molecular electrostatic potential revealed the sites prone to electrophilic and nucleophilic attacks in meropenem.

## Competing interests

The authors declare that they have no competing interests.

## Authors’ contributions

JCP formulated the research idea and planned the experiment, carried out characterization and interpretation results and wrote the manuscript, MP carried out UV spectra, KL prepared FT-IR and Raman spectra and interpreted them, BB carried out theoretical calculations and PZ, PG prepared the figures and tables. All the authors read and approved the final manuscript.
